# Association of temporomandibular disorder-related pain with severe headaches—a Bayesian view

**DOI:** 10.1007/s00784-021-04051-y

**Published:** 2021-07-05

**Authors:** Javed Ashraf, Matti Närhi, Anna Liisa Suominen, Tuomas Saxlin

**Affiliations:** 1 Institute of Dentistry, University of Eastern Finland, P.O. Box 1627, 70211 Kuopio, Finland; 2 Institute of Biomedicine, University of Eastern Finland, Kuopio, Finland; 3 Department of Oral and Maxillofacial Diseases, Kuopio University Hospital, Kuopio, Finland; 4 Department of Public Health and Welfare, the Finnish Institute for Health and Welfare, Helsinki, Finland

**Keywords:** Temporomandibular disorders, Headache disorders, Pain, Bayesian logistic regression, Sensitivity analysis, Directed acyclic graphs

## Abstract

**Objectives:**

Association of temporomandibular disorders (TMD)-related pain with severe headaches (migraine and tension-type headaches [TTH]) was studied over a follow-up period of 11 years.

**Materials and methods:**

The data used was from two nationally representative health surveys in Finland—the Health 2000 Survey (baseline) and the Health 2011 Survey (follow-up) (Bioresource Research Impact Factor [BRIF] 8901)—conducted by the Finnish Institute for Health and Welfare (THL). The primary dataset of the current study included a subset of the population undergoing a clinical oral examination, including TMD examination, at baseline, and answering the questions related to severe headaches, both at baseline and at follow-up (n = 530). From the primary dataset, two datasets were created to study the onset of migraine (dataset 1) and TTH (dataset 2) separately. Dataset 1 included participants healthy of migraine, but not other headaches, at baseline (n = 345), and dataset 2 participants healthy of TTH and other headaches, except migraine, at baseline (n = 464). Bayesian logistic regression models with weakly informative priors were utilized to assess the association of muscle-related TMD pain (mTMD) at baseline and temporomandibular joint-related TMD pain (jTMD) at baseline with the presence of migraine and TTH at follow-up.

**Results:**

Neither of the baseline TMD-related pain variables were associated with the presence of migraine at follow-up (posterior effect estimates-0.12, 95% credible interval [CI] -0.49–0.24, and 0.11, 95% CI -0.38–0.59, for mTMD and jTMD, respectively), whereas mTMD at baseline (posterior effect estimate 0.36, 95% CI 0.02–0.69), but not jTMD at baseline (posterior effect estimate -0.32, 95% CI -0.94–0.25), was associated with the presence of TTH at follow-up. Bayesian sensitivity analyses revealed that the estimates of the regression models were stable, demonstrating sufficient validity and consistency of the estimates.

**Conclusion:**

These results indicate that diverse mechanisms may exist behind the associations of TMD-related painful conditions with different types of severe headaches.

**Clinical relevance:**

TMD-related pain is a frequent comorbidity of severe primary headaches. Therapy of severe primary headaches may thus benefit significantly with the incorporation of a multi-disciplinary clinical team.

## Introduction

The prevalence of temporomandibular disorder (TMD) symptoms varies significantly between populations. A recent systematic review indicated that in general populations the prevalence of having at least one clinical sign of TMD ranges between 5 and 60% [1]. Nonetheless, pain in the temporomandibular region is a common clinical sign, occurring in approximately 10% of the adult population [2]. In Finland, a recent study reported that at least one-third (34.6%) of the Finnish population suffered from at least one clinical sign of TMD. This study also reported that the prevalence of muscle-related TMD pain (mTMD) was 1.9% and 6.5% for males and females, and the prevalence of temporomandibular joint-related TMD pain (jTMD) was 1.7% and 3.5% for males and females, respectively [3].

Primary headaches (migraine and tension-type headaches [TTH]), on the other hand, affect more than 2.5 billion individuals worldwide. A recent global study ranked headaches as the second leading cause of years lost due to disability after lower back pain [4]. Globally, the number of individuals suffering from migraine and TTH in the year 2017 was estimated to be 1.3 and 2.3 billion, and the percentage of increase of those suffering from these disorders within the decade 2007–2017 was 15% and 16%, respectively [5]. In Finnish population—according to a recent retrospective study based on the electronic medical records of Finland’s largest private occupational health care provider—the overall prevalence of migraine was 7% in females and 2% in males [6]. Moreover, the proportion of TTH sufferers in Finland has been amounted to 16% of the total population [7].

TMD-related pain (mTMD and jTMD) seldom occurs in isolation; approximately only 17% of the TMD-related pain cases occur with no other comorbid pain condition [8]. TMD-related pain is often associated with other chronic pains causing significant physical and psychological disability [9]. Amongst the comorbidities of the TMD-related pain, headaches—such as migraine and TTH—are frequently reported along with both mTMD and jTMD [10]. Association of mTMD with migraine has been reported also in a recent cross-sectional study based on a representative study sample of adult Finnish population [11].

To the best of the authors’ knowledge, all the previous studies of the association of TMD-related pain with headaches have been based on the Frequentist statistics. Compared to the Bayesian approach, the Frequentist statistics suffer from some limitations, most importantly the dependence on large sample sizes for effect sizes to be accurately determined [12]. Additionally, in contrast to the Frequentist methodology, the Bayesian statistics do not provide one (fixed) outcome value but rather an interval containing the regression coefficient [13]. These intervals, termed credible intervals (CI), attribute a probability to the best estimate and to all the possible values of the parameter estimates [12].

The current study—utilizing the Bayesian methodology—aimed to examine the association of TMD-related pain with severe headaches (migraine and TTH) over a follow-up period of 11 years. The hypothesis was that a prospective association exists between TMD-related pain and severe headaches.

## Materials and methods

### Study sample and participants

The current study utilized data from the Health 2000 (baseline) and the Health 2011 (follow-up) Surveys (Bioresource Research Impact Factor [BRIF] 8901), conducted by the Finnish Institute for Health and Welfare (THL) (former National Public Health Institute [KTL] of Finland).

The Health 2000 Survey, conducted in the years 2000 and 2001, included 9922 invited participants aged 18 years or older living in mainland Finland. The data for this survey were collected through self-administered questionnaires and interviews, and for those aged 30 years or older also by clinical oral and health examinations as well as by laboratory analyses. The participation rate of the Health 2000 Survey was 92% (*n* = 9125) (participation in at least one phase of the survey) [14].

The Health 2011 Survey was a follow-up study of the Health 2000 Survey, fieldwork of which was conducted in the years 2011–2012. The invited participants of the Health 2011 Survey included those participants of the Health 2000 Survey sample who were alive, living in Finland, had contact details available, and not refused to participate in further surveys previously (*n* = 8135), participation rate in at least one phase of the survey being 73% (*n* = 5903). The main reason for non-participation was the refusal to participate, following inability to contact the participant, the death of the invited participant, and the invited participant living abroad. The Health 2011 Survey included many smaller studies specific to a common disorder/group of disorders. Participants reporting to ever suffer from severe (moderate to intense) headaches during the health interview of the Health 2011 Survey proper (*n* = 875) were invited to participate one of the smaller studies, namely Migraine Sub-study. It included questions on headache types, severity, and their impact. Of the eligible participants, 832 eventually participated (95%) [15].

**Fig. 1:**
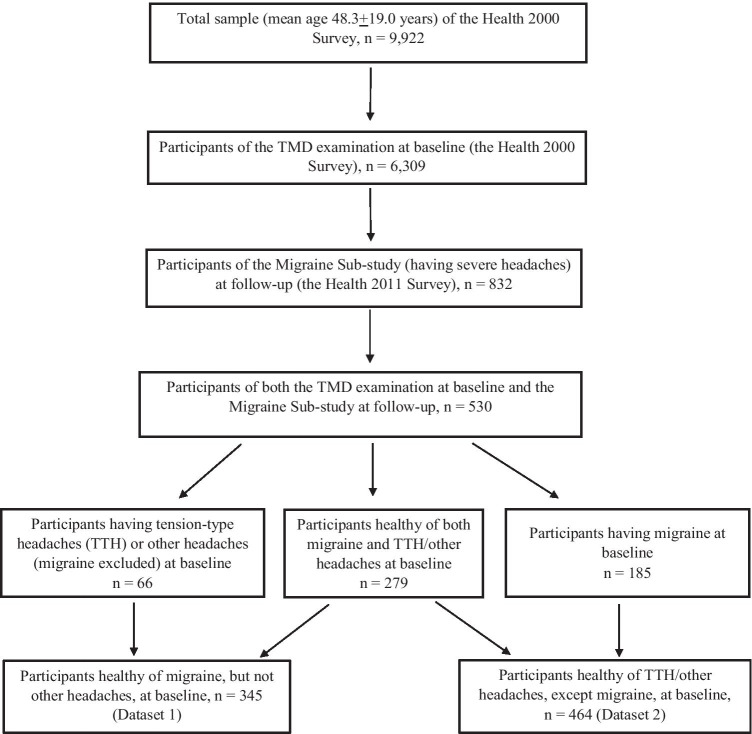
Schematic representation of the sample utilized in the study

The current study utilized a subset of the population undergoing clinical oral examination, including TMD examination, at baseline (the Health 2000 Survey) and answering the questions related to severe headaches both at baseline and at follow-up (the Migraine Sub-study of the Health 2011 Survey) (*n* = 530). From this subset, two datasets were created to study the onset of migraine (dataset 1) and TTH (dataset 2), and were analyzed separately. Dataset 1 included only the participants healthy of migraine, but not other headaches, at baseline (reported in the interview of not having migraine diagnosed by a physician in the Health 2000 Survey) (*n* = 345), and dataset 2 only the participants not suffering from TTH or other headaches, except migraine, at baseline (reported in the interview of not having TTH or other headaches [migraine excluded] diagnosed by a physician in the Health 2000 Survey) (*n* = 464) (Fig. 1).

### Outcome variables

The outcome variables were the presence (yes/no) of migraine or TTH at follow-up (the Health 2011 Survey). These were asked by questions: “Did a physician ever diagnose you for migraine?” and “Did a physician ever diagnose you for tension-type headaches?” [15].

### Predictor variables

In the Health 2000 Survey, five experienced and calibrated dentists performed a standardized clinical oral examination. This included TMD examination partly based on the Research Diagnostic Criteria for TMD (RDC-TMD) Axis I criteria [16], focusing on the most central clinical aspects. The TMD examination involved the palpation of the masticatory muscles (*m. temporalis* and *m. masseter*) and the temporomandibular joint (TMJ). The pain response during the inspection of the temporomandibular area was recorded dichotomously (yes/no). Further details regarding the TMD examination methodology and protocol can be found at Suominen-Taipale et al. (2008). TMD-related pain variables, mTMD (yes/no) and jTMD (yes/no), were used as the predictor variables. The percentage of agreement for recording pain responses during the TMD examination between the examiners and the reference examiner was 95% (kappa value 0.47; 95% CI 0.41–0.53) for mTMD and 92% (kappa value 0.26; 95% CI 0.19–0.34) for jTMD [17].

### Other covariates

Other covariates and potential confounders of the current study were utilized from the baseline data (the Health 2000 Survey). These included gender and age taken from the population registers. Variables recorded during the home-visit interview included the level of education (education below upper secondary or vocational school level [low]; graduated from upper secondary school or vocational school [medium]; and graduated from a university or a polytechnic institute [high]), and the use of anti-inflammatory drugs (yes/no). Body mass index (BMI) was calculated using the weight and height of the study participants assessed during the health examination (if not available, information from a questionnaire was used). BMI was used as a continuous variable in the analyses [18].

### Statistical analyses

Differences in the frequencies of the categories of the predictor variables and the categorized covariates between those participants reporting *vs.* those not reporting migraine at follow-up, as well as between the TTH and non-TTH study participants at follow-up, were analyzed using chi-square test. Regarding the continuous covariates, the normality of the distributions in different categories of the outcome variables was assessed visually through distribution histograms and tested with Kolmogorov–Smirnov and Shapiro–Wilk tests. Based on these, the Mann–Whitney U test was used to observe differences amongst them in the different outcome variable categories. The p-values for the descriptive statistics of the study population were based on two-tailed tests of significance.

**Fig. 2:**
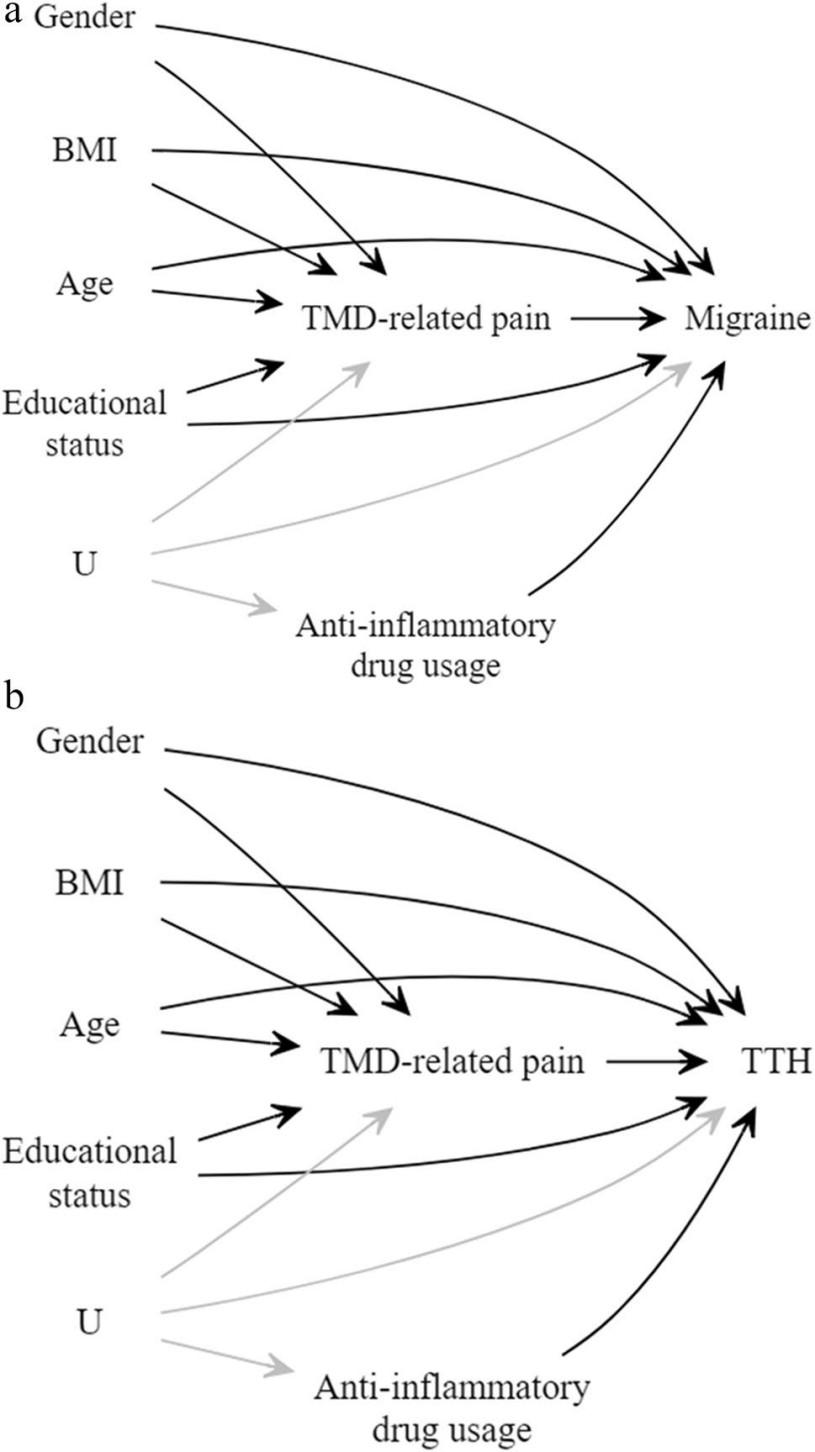
**a** Directed acyclic graphs depicting the hypothetical model for the associations between TMD-related pain, covariates, and the presence of migraine. U, unmeasured confounder, *i.e.*, psychological status. Grey arrows = denoting disjunctive cause between the outcome variable and the covariate of anti-inflammatory drug usage through unmeasured confounder of psychological status. **b** Directed acyclic graphs depicting the hypothetical model for the associations between TMD-related pain, covariates, and the presence of tension-type headaches (TTH). U, unmeasured confounder, *i.e.*, psychological status. Grey arrows = denoting disjunctive cause between the outcome variable and the covariate anti-inflammatory drug usage through unmeasured confounder of psychological status

The current study employed directed acyclic graphs (DAG) for the evaluation of the study hypothesis, based on a priori knowledge (Fig. 2a and b). Associations of the potential confounders and covariates with either or both the predictor and the outcome variables in the DAG models were adjusted for in the regression models. These adjustments were made through blocking all the backdoor paths and implementing the disjunctive cause criterion in the DAG [19].

Multivariate Bayesian logistic regression analyses were performed for analyzing the effect estimates between the predictor and the outcome variables. Weakly informative priors (a normal distribution with the mean of zero and the variance of five) were utilized for the regression models in both datasets due to the lack of substantial longitudinal evidence regarding the association of TMD-related pain with severe headaches. Another reason was the better computational stability of the Bayesian algorithm incorporating weakly informed priors in the case of logistic regression models [20]. The effect sizes of the association of TMD-related pain with severe headaches were estimated using predictive (regression) models. All the analyses of the current study reported the posterior effect estimates (regression coefficients; mean parameter value μ) with their Bayesian 95% credible intervals (CI). Bayesian sensitivity analyses were performed with varying prior distributions for the predictor variables to check for the reliability and stability of the parameter estimate distributions. Visual inspection of the estimation convergence of all regression models was done through graphical summaries of Bayesian parameter trace plots, Bayesian posterior parameter distribution plots, and Bayesian autocorrelation plots. A posterior predictive p-value of > 0.05 was taken as a good model fit for all the regression models. Complete case analyses was adhered to in the regression analyses; the missing data was handled by excluding all the study participants with incomplete data from the analyses.

SPSS (IBM Corp., Armonk, NY, USA) version 27 was used for the descriptive data analyses. Bayesian regression and sensitivity analyses were performed through Mplus version 8.4 (Muthen & Muthen, 1998–2019, Los Angeles, CA, USA). DAGs and flow charts for the current study were drawn through DigrammeR package of R software (http://www.R-project.org/).

### Ethical issues

Both surveys were based on voluntariness, and all the participants gave their written informed consent before their participation. Ethical approval for the Health 2000 Survey was obtained from the Ethics Committee for Epidemiology and Public Health of the Hospital District of Helsinki and Uusimaa, Finland. Ethical approval for the Health 2011 Survey was obtained from the Coordinating Ethics Committee at the Hospital District of Helsinki and Uusimaa, Finland [15, 18].

Guideline from the STROBE Statement—checklist was adhered to in the current manuscript.

## Results

Basic characteristics of the participants with (*n* = 269) and without (*n* = 261) migraine at follow-up, and participants with (*n* = 105) and without TTH (*n* = 425) at follow-up are presented in Tables 1 and 2, respectively.

**Table 1:** Characteristics of the study participants at baseline (the Health 2000 Survey) by the presence of migraine at follow-up (Health 2011 Survey), dataset 1, n = 345

	Presence of migraine at follow-up			
	Yes	No	Total, n (%)	*p*-value
Muscle-related TMD pain, n (%)				1.00*
Yes	22 (19.5)	46 (19.8)	68 (19.7)	
No	91 (80.5)	186 (80.1)	277 (80.3)	
Total	113 (100)	232 (100)	345 (100)	
Joint-related TMD pain, n (%)				0.55*
Yes	12 (10.6)	19 (8.2)	31 (9.0)	
No	101 (80.4)	213 (91.8)	314 (91.0)	
Total	113 (100)	232 (100)	345 (100)	
Gender, n (%)				< 0.01*
Males	20 (17.7)	92 (39.7)	112 (32.5)	
Females	93 (82.3)	140 (60.3)	233 (67.5)	
Total	113 (100)	232 (100)	345 (100)	
Age, median (SD)	44.4 (10.6)	46.6 (11.7)		0.14^†^
Education level, n (%)				0.39*
Low	28 (24.8)	53 (22.8)	81 (23.5)	
Medium	34 (30.1)	87 (37.5)	121 (35.1)	
High	51 (45.1)	92 (39.7)	143 (41.4)	
Total	113 (100)	232 (100)	345 (100)	
BMI, mean (SD)	26.0 (4.5)	26.1 (4.0)		0.89^†^
Use of anti-inflammatory drugs, n (%)				0.03*
Yes	63 (58.9)	102 (46.4)	165 (50.5)	
No	44 (41.1)	118 (53.6)	162 (49.5)	
Total	107 (100)	220 (100)	327 (100)	

^*^p-value calculated through chi-square test

^†^*p*-value calculated through Mann–Whitney U test

**Table 2:** Characteristics of the study participants at baseline (the Health 2000 Survey) by the presence of tension-type headaches (TTH) at follow-up (Health 2011 Survey), dataset 2, n = 464

	Presence of TTH at follow-up			
	Yes	No	Total, n (%)	*p*-value
Muscle-related TMD pain, n (%)				0.05*
Yes	24 (27.3)	66 (17.6)	90 (19.4)	
No	64 (72.7)	310 (82.4)	374 (80.6)	
Total	88 (100)	376 (100)	464 (100)	
Joint-related TMD pain, n (%)				0.48*
Yes	4 (4.5)	28 (7.4)	32 (6.9)	
No	84 (95.5)	348 (92.6)	432 (93.1)	
Total	88 (100)	376 (100)	464 (100)	
Gender, n (%)				0.01*
Males	14 (15.9)	111 (29.5)	125 (26.9)	
Females	74 (84.1)	265 (70.5)	339 (73.1)	
Total	88 (100)	376 (100)	464 (100)	
Age, mean (SD)	44.3 (9.2)	46.0 (10.8)		0.31^†^
Education level, n (%)				< 0.01*
Low	9 (10.2)	92 (24.5)	101 (21.8)	
Medium	23 (26.1)	132 (35.1)	155 (33.4)	
High	56 (63.6)	152 (40.4)	208 (44.8)	
Total	88 (100)	376 (100)	464 (100)	
BMI, mean (SD)	25.5 (4.8)	26.1 (4.1)		0.08^†^
Use of anti-inflammatory drugs, n (%)				0.47*
Yes	51 (60.0)	200 (54.9)	251 (55.9)	
No	34 (40.0)	164 (45.1)	198 (44.1)	
Total	85 (100)	364 (100)	449 (100)	

^*^p-value calculated through chi-square test

^†^*p*-value calculated through Mann–Whitney U test

In the regression analyses of the dataset 1—after adjusting for potential confounding factors such as gender, age, the level of education, BMI, and the use of anti-inflammatory drugs—the 95% CI of the posterior effect estimates of both mTMD and jTMD at baseline in the association with the presence of migraine at follow-up included null values, thus denoting no association between these variables (Table 3).

**Table 3:** Bayesian logistic regression analyses of the association of TMD-related pain (mTMD and jTMD) with the presence of migraine and tension-type headaches (TTH) at follow-up

Predictor	Posterior effect estimate (SD^†^)	95% credible interval	Posterior predictive *p*-value
Dataset 1^‡^ (effective n = 327)			
mTMD	-0.12 (0.19)	-0.49–0.24	0.20
jTMD	0.11 (0.25)	-0.38–0.59	0.34
Dataset 2^§^ (effective n = 449)			
mTMD	0.36 (0.17)	0.02–0.69	0.13
jTMD	-0.32 (0.30)	-0.94–0.25	0.16

Adjusted for gender, age, the level of education, body mass index (continuous), and the use of anti-inflammatory drugs

^†^Standard deviation

^‡^Migraine at follow-up as the dependent variable

^**§**^TTH at follow-up as the dependent variable

According to the regression analyses of the dataset 2, after adjusting for confounding factors, mTMD at baseline was found to predict the presence of TTH at follow-up (posterior effect estimate 0.36, 95% CI 0.02–0.69). However, null value was included in the 95% CI of the posterior effect estimate of jTMD at baseline in the association with the presence of TTH at follow-up suggesting no association (Table 3).

All the regression models displayed a posterior predictive p-value > 0.05, suggesting an acceptable fit of the models (Table 3). Additionally, all the regression models demonstrated good convergence, low autocorrelation, and near-to-normal posterior distributions upon visual inspections of the Bayesian posterior parameter trace plots, Bayesian autocorrelation plots, and Bayesian posterior parameter distributions, respectively. According to the Bayesian sensitivity analyses, the effect estimates of all the regression models were stable, demonstrating sufficient validity and reliability of the effect estimates. The sensitivity analyses depicted less than 1% change in the posterior effect estimates upon the introduction of different prior ranges for the predictor variables (Table 4).

**Table 4:** Sensitivity analyses of the association of TMD-related pain (mTMD and jTMD) with the presence of migraine and tension-type headaches (TTH) at follow-up

Predictor	Default priors^†^, N (0, 10^10^)	Current study priors, N (0, 5)^‡^	Testing priors, N (0, 10)	Testing priors, N (2, 10)
Dataset 1^§^ (effective n = 327)				
mTMD				
Posterior effect estimate (SD)	-0.12 (0.19)	-0.12 (0.19)	-0.12 (0.19)	-0.12 (0.19)
jTMD				
Posterior effect estimate (SD)	0.11 (0.25)	0.11 (0.25)	0.11 (0.25)	0.12 (0.25)
Dataset 2^¶^ (effective n = 449)				
mTMD				
Posterior effect estimate (SD)	0.36 (0.17)	0.36 (0.17)	0.36 (0.17)	0.37 (0.17)
jTMD				
Posterior effect estimate (SD)	-0.32 (0.30)	-0.32 (0.30)	-0.32 (0.30)	-0.30 (0.30)

Adjusted for gender, age, the level of education, body mass index (continuous), and the use of anti-inflammatory drugs

^**†**^Default (non-informative) priors of the software MPlus version 8.4

^‡^Refers to a prior with a normal distribution, mean of zero, and variance of five

^**§**^Migraine at follow-up as the dependent variable

^¶^TTH at follow-up as the dependent variable

## Discussion

The current study reported that mTMD at baseline predicts the presence of TTH at follow-up. However, no consistent association was found between jTMD at baseline and the presence of TTH at follow-up. In addition, neither of the TMD-related pain variables at baseline were found to be associated with the presence of migraine at follow-up.

A key strength of the current study is the measurement of the predictor variables mTMD and jTMD through clinical TMD examination with good inter-examiner reliability. Clinical examination adds to the measurement validity of the TMD-related pain variables, hence increasing the reliability of the effect estimates. A follow-up period of 11 years can be considered a strength in certain respect, namely justifying a sufficient effect of the predictors on the conditions with a slow onset, such as severe headaches. The utilization of DAGs for the appropriate selection of covariates/confounders to be entered in the regression models can also be considered a strength of the current study. DAGs aid in determining the unbiased  associations between the predictor and the outcome variables. Another strength of the study is depicted through application of the Bayesian sensitivity analyses, which demonstrated the stability of the parameter estimates between the variables. This stability measure denotes a sufficient reliability of the parameter estimates of the models. Furthermore, the posterior distribution of the effect estimates places higher credibility on parameter values that are more consistent with the data, *i.e.*, more stable [21].

The current study naturally has also limitations. An obvious shortcoming is the lack of psychosocial aspect (the RDC/TMD Axis II criteria) in the TMD examination. However, this was taken into consideration by adding the anti-inflammatory drug usage as a covariate, since a correlation has been reported between the anti-inflammatory drug usage and the psychological status of migraine [22] and TTH [23] patients. In addition, although the long follow-up period has merits, the lack of measurement time points during the follow-up period must be considered a limitation. The remitting and recurring nature of both TMD-related pain and severe headaches may have caused fluctuations possibly biasing the parameter estimates, a problem not so obvious with more stable exposures and outcomes. The outcome variables assessed by self-report, although being based on a physician diagnosis of severe headaches, can also be considered a limitation. For instance, due to the lengthy follow-up period, recall bias may arise of participants not remembering their diagnoses correctly in the case of rare, intermittent headaches. Moreover, the dichotomous nature of the outcome variables, as well as the low proportion of chronic migraineurs in the current study sample, may also be regarded as limitations of the current study. One possible source of bias may also arise from the overlap between mTMD and TTH; however, the effect of this should minimal, since all the participants with TTH at baseline were excluded and only 7% of the TTH sufferers at follow-up had both mTMD and TTH. Lastly, although thorough statistical inferential techniques for robust transparency of the results were utilized, these effect estimates could not be interpreted beyond the causal association paradigm.

The current study is, according to the authors’ knowledge, the first prospective study reporting the effect estimates between different TMD-related pain variables and the presence of migraine and TTH through Bayesian methodology. Bayesian methodology, which compared to its Frequentist counterpart, provides better estimations in situations of non-random or nested study samples [24]. Utilization of the Bayesian sensitivity analyses also allows for a transparent, yet robust, assessment of the validity and reliability of the effect estimates.

The Bayesian methodology employed in the present study offers several advantages over the traditional Frequentist methodology. This analytical approach produces a range of values (Bayesian 95% CI) as posterior distributions that reflect the uncertainty inherent for the multiple unknown parameters, rather than a fixed value for parameter estimates as in Frequentist approaches. Also, the Bayesian statistical analyses allow for the sub-analyses without the need for the classic statistical adjustments for multiple comparisons. The Bayesian parameter estimates may remain less biased and hence more appropriate and stable with moderate and even smaller sample sizes [25]. Additionally, the interpretation employed to define 95% confidence intervals in the Frequentist statistics is, in fact, a Bayesian definition for the 95% CI estimation. The interpretation of the Bayesian 95% CI states a 95% probability that the population parameter lies within its defined range [21]. Bayesian methodology should, therefore, be utilized on a more regular basis for better and unbiased effect estimates, especially with smaller or partially non-random nested samples, such as in the current study. As fully informative priors could not be utilized in the current study, Bayesian sensitivity analyses aided in placing meaningful boundaries on the conclusions. These boundaries are needed when there is uncertainty in the choice of priors [26].

The current study found no association between TMD-related pain (mTMD and jTMD) at baseline and the presence of migraine at follow-up. Only one study [27], to our knowledge, has reported a prospective association between TMD-related pain and migraine. However, that study differed from this study in several aspects such as having a distinctly smaller and selected sample of participants in a tertiary health care facility *vs.* population-based sample in this study. Another difference was the clearly larger proportion of chronic migraineurs in the study population of that study compared to the current study population (52.3% *vs*. 1.8%, respectively). Increased pain sensitivity and lowered pressure pain thresholds have been reported along with higher frequency of migraine headaches [28]. These differences may explain the contrasting association patterns between the current study and the study by Stuginski-Barbosa et al. (2010).

The prospective association of mTMD at baseline with the presence of TTH at follow-up found in the current study is in line with previous epidemiological, clinical, and physiological evidence. Previous epidemiological studies have shown an association between TMD-related pain and TTH [29]. Clinically, TMD-related pain and TTH share a combination of distinct signs and symptoms in the head and face region, particularly evident as regards to mTMD and TTH. These common clinical features include palpation tenderness of the masticatory muscles in the case of mTMD and the pericranial muscles in the case of TTH during the active phases of both conditions [30]. Other clinical intersections between mTMD and TTH include subjects’ age regarding the peak prevalence [31], the intensity of pain, pharmacotherapy [32], and even the non-pharmacological treatment [33]. In spite of some clinical similarities and overlap, both mTMD and TTH are distinct disease entities. Although the mix of similarities may necessitate a close interdisciplinary co-operation between specialties (dentistry *vs*. neurology), vigilance should also be exercised regarding the distinction between these two disease entities during their treatment.

In terms of autonomic dysfunction, both mTMD [34] and TTH [35] utilize the trigeminal system to relay nociceptive afferents to their respective higher brain centers. Features shared by mTMD and TTH also include the lower pressure pain threshold and referred pain during their active phases [34, 35]. These features reflect the presence of peripheral and central sensitization in their respective trigeminal nociceptive pathways. A recent study also reported a high prevalence of active myofascial trigger points in TTH patients [36]. This finding may support the hypothesis that peripheral muscular mechanisms are involved in the pathophysiology of TTH.

Current research reports a significant role of central sensitization in the pathogenesis of chronic TTH [37]. Continuous painful episodes involving pericranial muscles (such as the temporal muscle) may hypersensitize the central nervous system activating the higher brain centers leading to chronic transformation of TTH [38]. This role of mTMD in the chronicity of TTH should always be considered by the dentists and neurologists while treating mTMD and TTH, respectively.

The finding of no association between jTMD and TTH in the current study is in accordance with the findings of a cross-sectional study by Gonçalves et al. (2011) [39]. This may be due to mTMD being more of a generalized pain condition with central sensitization as an important component of its pathogenesis [40], like with the pathogenesis of TTH [29]. Unlike mTMD, jTMD has been considered a localized pathology [41], thus perhaps having a minor effect on the pathogenesis of TTH.

## Conclusions

The current study reported a prospective association of mTMD, but not jTMD, with the presence of TTH. However, TMD-related pain was not found to be associated with the presence of migraine. These results were found reliable after the application of Bayesian sensitivity analyses. An interpretation of these results may be the existence of diverse mechanisms playing a role in the associations between different TMD-related painful conditions and the presence of severe headaches.
